# Aqua­bis(dichloro­acetato-κ*O*)(1,10-phenanthroline-κ^2^ *N*,*N*′)copper(II)

**DOI:** 10.1107/S1600536808042578

**Published:** 2008-12-20

**Authors:** Yaru Liu, Jianzhong Ning, Junshan Sun, Chuan Zhang

**Affiliations:** a School of Science, North University of China, Taiyuan 030051, People’s Republic of China; b College of Chemistry and Food, Zhongzhou University, Zhengzhou 450044, People’s Republic of China; c Department of Materials Science and Chemical Engineering, Taishan University, 271021 Taian, Shandong, People’s Republic of China

## Abstract

In the title complex, [Cu(C_2_HCl_2_O_2_)_2_(C_12_H_8_N_2_)(H_2_O)], the Cu^II^ ion has a distorted square-pyramidal coordination geometry. The equatorial positions are occupied by two N atoms from a 1,10-phenanthroline ligand [Cu—N = 1.994 (3) and 2.027 (3) Å] and two O atoms from dichloro­acetate ligands and a water mol­ecule [Cu—O = 1.971 (2) and 1.939 (2) Å]. One O atom from another dichloro­acetate ligand occupies the apical positon [Cu—O = 2.152 (3) Å]. Inter­molecular O—H⋯O hydrogen bonds link the mol­ecules into centrosymmetric dimers. The crystal packing also exhibits weak inter­molecular C—H⋯O hydrogen bonds, π–π inter­actions [centroid–centroid distance = 3.734 (2) Å] and short inter­molecular Cl⋯Cl contacts [3.306 (2) and 3.278 (2) Å].

## Related literature

For applications of dichloro­acetic acid derivatives, see: Múdra *et al.* (2003 ▶); Lin *et al.* (2001 ▶); Zhu & Xiao (2006 ▶).
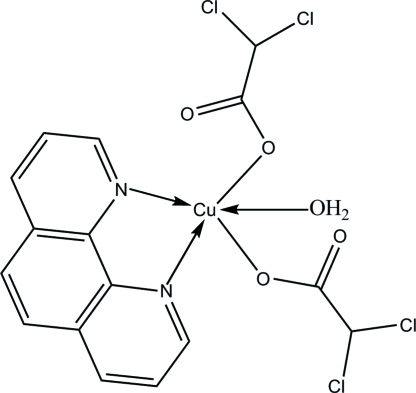


## Experimental

### 

#### Crystal data

[Cu(C_2_HCl_2_O_2_)_2_(C_12_H_8_N_2_)(H_2_O)]
*M* *_r_* = 517.62
Triclinic, 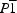

*a* = 8.2701 (8) Å
*b* = 10.8883 (11) Å
*c* = 12.0125 (12) Å
α = 67.4390 (10)°
β = 77.585 (2)°
γ = 73.776 (2)°
*V* = 952.02 (16) Å^3^
*Z* = 2
Mo *K*α radiation
μ = 1.74 mm^−1^
*T* = 273 (2) K
0.32 × 0.25 × 0.21 mm

#### Data collection

Bruker SMART CCD area-detector diffractometer
Absorption correction: multi-scan (*SADABS*; Sheldrick, 1996 ▶) *T*_min_ = 0.606, *T*_max_ = 0.711
5043 measured reflections
3346 independent reflections
2539 reflections with *I* > 2σ(*I*)
*R*_int_ = 0.064

#### Refinement

*R*[*F* ^2^ > 2σ(*F* ^2^)] = 0.042
*wR*(*F* ^2^) = 0.106
*S* = 1.01
3346 reflections
261 parameters
3 restraints
H atoms treated by a mixture of independent and constrained refinement
Δρ_max_ = 0.63 e Å^−3^
Δρ_min_ = −0.51 e Å^−3^

### 

Data collection: *SMART* (Siemens, 1996 ▶); cell refinement: *SAINT* (Siemens, 1996 ▶); data reduction: *SAINT*; program(s) used to solve structure: *SHELXS97* (Sheldrick, 2008 ▶); program(s) used to refine structure: *SHELXL97* (Sheldrick, 2008 ▶); molecular graphics: *SHELXTL* (Sheldrick, 2008 ▶); software used to prepare material for publication: *SHELXTL*.

## Supplementary Material

Crystal structure: contains datablocks I, global. DOI: 10.1107/S1600536808042578/cv2493sup1.cif

Structure factors: contains datablocks I. DOI: 10.1107/S1600536808042578/cv2493Isup2.hkl

Additional supplementary materials:  crystallographic information; 3D view; checkCIF report

## Figures and Tables

**Table 1:** Hydrogen-bond geometry (Å, °)

*D*—H⋯*A*	*D*—H	H⋯*A*	*D*⋯*A*	*D*—H⋯*A*
C16—H16⋯O2^i^	0.98	2.24	3.118 (5)	149
O5—H5*B*⋯O2^ii^	0.85 (2)	1.81 (2)	2.654 (3)	174 (3)
O5—H5*A*⋯O4	0.85 (2)	1.86 (2)	2.673 (4)	159 (3)

Symmetry codes: (i) 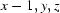; (ii) 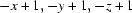.

**Fig. 1.**
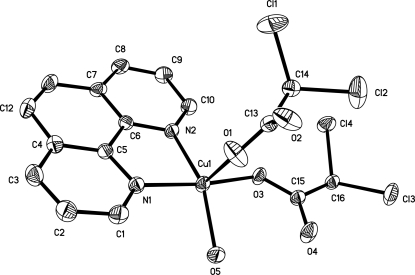
The structure of the title complex, showing 30% probability displacement ellipsoids and the atom-numbering scheme. The H atoms are omitted.

## supplementary crystallographic information

### Comment

Dichloroacetic acid and its derivatives are biologically active compounds which have been widely studied because of their fascinating topologies and potential applications as functional materials (Múdra *et al.*, 2003; Lin *et al.*, 2001; Zhu *et al.*, 2006;). In our study of this field, we selected 1,10-phenanthroline as the co-ligand to continue our exploration to the Cu complexes with the dichloroacetic acid ligand. Herein we report the structure of the title complex (I).

In (I) (Fig. 1), the Cu ion has a distorted square-pyramidal coordination. Two N atoms from 1,10-phenanthroline ligand and two oxygen atoms from a dichloroacetic acid ligand form a basal plane, and an aqua atom occupy the axial apical position. Intermolecular O—H···O hydrogen bonds (Table 2) link the molecules into centrosymmetric dimers. The crystal packing exhibits also weak intermolecular C—H···O hydrogen bonds, π–π interactions and short intermolecular Cl···Cl contacts (Table 1).

### Experimental

A mixture of Cu(CH3COO)2*3H2O(204 mg, 1 mmol) and 1,10-phenanthroline (185 mg, 1 mmol) in methanol(30 ml) was placed in a Teflon-lined stainless steel Parr bomb that was heated at 403 K for 48 h. The bomb was then cooled down to the room temperature, the solution was filtered. The solvent was removed from the filtrate under vacuum, and the solid residue was recrystallized from diethyl ether; blue crystals suitable for X-Ray diffraction study were obtained. Yield, 0.760 g, 83%. m.p. 573 K. Analysis, calculated for C_16_H_12_Cl_4_CuN_2_O_5_: C 46.73, H 2.94, N 6.81; found: C 46.95, H 2.56, N 7.07%. The elemental analyses were performed with a Perkine Elemer PE2400II instrument.

### Refinement

C-bound H atoms were geometrically positioned (C—H 0.93–0.97 Å) and refined as riding, with *U*_iso_(H)=1.2*U*_eq_(C). The water H atoms were located on a Fourier map and isotropically refined with the distance restraints O—H=0.85 (2) Å.

### Crystal data

**Table:**

[Cu(C_2_HCl_2_O_2_)_2_(C_12_H_8_N_2_)(H_2_O)]	*Z* = 2
*M**_r_* = 517.62	*F*(000) = 518
Triclinic, *P*1	*D*_x_ = 1.806 Mg m^−^^3^
*a* = 8.2701 (8) Å	Mo *K*α radiation, λ = 0.71073 Å
*b* = 10.8883 (11) Å	Cell parameters from 1923 reflections
*c* = 12.0125 (12) Å	θ = 2.3–27.1°
α = 67.439 (1)°	µ = 1.74 mm^−^^1^
β = 77.585 (2)°	*T* = 273 K
γ = 73.776 (2)°	Block, colorless
*V* = 952.02 (16) Å^3^	0.32 × 0.25 × 0.21 mm

### Data collection

**Table:**

Bruker SMART CCD area-detector diffractometer	3346 independent reflections
Radiation source: fine-focus sealed tube	2539 reflections with *I* > 2σ(*I*)
graphite	*R*_int_ = 0.064
φ and ω scans	θ_max_ = 25.1°, θ_min_ = 1.9°
Absorption correction: multi-scan (*SADABS*; Sheldrick, 1996)	*h* = −9→8
*T*_min_ = 0.606, *T*_max_ = 0.711	*k* = −12→12
5043 measured reflections	*l* = −14→14

### Refinement

**Table:**

Refinement on *F*^2^	Primary atom site location: structure-invariant direct methods
Least-squares matrix: full	Secondary atom site location: difference Fourier map
*R*[*F*^2^ > 2σ(*F*^2^)] = 0.042	Hydrogen site location: inferred from neighbouring sites
*wR*(*F*^2^) = 0.106	H atoms treated by a mixture of independent and constrained refinement
*S* = 1.00	*w* = 1/[σ^2^(*F*_o_^2^) + (0.046*P*)^2^] where *P* = (*F*_o_^2^ + 2*F*_c_^2^)/3
3346 reflections	(Δ/σ)_max_ < 0.001
261 parameters	Δρ_max_ = 0.63 e Å^−^^3^
3 restraints	Δρ_min_ = −0.51 e Å^−^^3^

### Special details

**Table:**

Geometry. All e.s.d.'s (except the e.s.d. in the dihedral angle between two l.s. planes) are estimated using the full covariance matrix. The cell e.s.d.'s are taken into account individually in the estimation of e.s.d.'s in distances, angles and torsion angles; correlations between e.s.d.'s in cell parameters are only used when they are defined by crystal symmetry. An approximate (isotropic) treatment of cell e.s.d.'s is used for estimating e.s.d.'s involving l.s. planes.
Refinement. Refinement of *F*^2^ against ALL reflections. The weighted *R*-factor *wR* and goodness of fit *S* are based on *F*^2^, conventional *R*-factors *R* are based on *F*, with *F* set to zero for negative *F*^2^. The threshold expression of *F*^2^ > σ(*F*^2^) is used only for calculating *R*-factors(gt) *etc*. and is not relevant to the choice of reflections for refinement. *R*-factors based on *F*^2^ are statistically about twice as large as those based on *F*, and *R*- factors based on ALL data will be even larger.

### Fractional atomic coordinates and isotropic or equivalent isotropic displacement parameters (Å^2^)

**Table:**

	*x*	*y*	*z*	*U*_iso_*/*U*_eq_	
Cu1	0.23680 (6)	0.74569 (4)	0.41387 (4)	0.03375 (16)	
Cl4	0.12884 (15)	0.83880 (10)	0.01776 (9)	0.0498 (3)	
Cl3	0.18005 (19)	0.55281 (11)	0.05837 (10)	0.0653 (4)	
Cl1	0.66991 (17)	0.90846 (12)	0.08639 (11)	0.0717 (4)	
Cl2	0.5908 (2)	0.69108 (14)	0.04161 (11)	0.0831 (5)	
O3	0.1567 (3)	0.7463 (2)	0.2733 (2)	0.0399 (6)	
C5	0.2814 (4)	0.9060 (3)	0.5379 (3)	0.0310 (8)	
O5	0.1749 (4)	0.5698 (3)	0.5130 (2)	0.0471 (7)	
N2	0.1747 (4)	0.9522 (3)	0.3541 (2)	0.0324 (7)	
N1	0.3082 (4)	0.7741 (3)	0.5489 (3)	0.0343 (7)	
C9	0.0716 (5)	1.1808 (4)	0.2276 (3)	0.0447 (10)	
H9	0.0263	1.2392	0.1564	0.054*	
O2	0.7530 (4)	0.6097 (3)	0.2708 (2)	0.0586 (8)	
C13	0.6067 (5)	0.6806 (4)	0.2683 (3)	0.0344 (8)	
C16	0.0957 (5)	0.6794 (3)	0.1249 (3)	0.0340 (8)	
H16	−0.0272	0.6861	0.1432	0.041*	
C11	0.2127 (6)	1.1856 (4)	0.4993 (4)	0.0471 (10)	
H11	0.1873	1.2779	0.4887	0.056*	
C15	0.1639 (5)	0.6447 (3)	0.2447 (3)	0.0341 (8)	
O4	0.2088 (4)	0.5243 (3)	0.3049 (2)	0.0609 (9)	
C14	0.5542 (5)	0.7783 (4)	0.1437 (3)	0.0357 (9)	
H14	0.4330	0.8198	0.1536	0.043*	
C1	0.3872 (5)	0.6806 (4)	0.6436 (3)	0.0447 (10)	
H1	0.4132	0.5893	0.6509	0.054*	
C12	0.2859 (6)	1.0935 (4)	0.5985 (4)	0.0510 (11)	
H12	0.3130	1.1242	0.6531	0.061*	
C8	0.1025 (5)	1.2327 (4)	0.3051 (3)	0.0421 (10)	
H8	0.0766	1.3264	0.2879	0.051*	
C10	0.1075 (5)	1.0398 (4)	0.2542 (3)	0.0398 (9)	
H10	0.0836	1.0063	0.2004	0.048*	
C6	0.2062 (5)	1.0040 (3)	0.4314 (3)	0.0306 (8)	
C7	0.1742 (5)	1.1436 (4)	0.4118 (3)	0.0368 (9)	
C4	0.3223 (5)	0.9501 (4)	0.6208 (3)	0.0390 (9)	
C2	0.4306 (6)	0.7160 (4)	0.7297 (4)	0.0506 (11)	
H2	0.4810	0.6482	0.7955	0.061*	
O1	0.5012 (4)	0.6801 (3)	0.3559 (2)	0.0634 (9)	
C3	0.4009 (6)	0.8478 (4)	0.7199 (3)	0.0477 (10)	
H3	0.4321	0.8710	0.7779	0.057*	
H5A	0.192 (6)	0.535 (3)	0.458 (2)	0.065 (16)*	
H5B	0.194 (6)	0.509 (3)	0.5816 (15)	0.068 (15)*	

### Atomic displacement parameters (Å^2^)

**Table:**

	*U*^11^	*U*^22^	*U*^33^	*U*^12^	*U*^13^	*U*^23^
Cu1	0.0471 (3)	0.0207 (2)	0.0318 (3)	−0.00140 (19)	−0.0107 (2)	−0.00826 (19)
Cl4	0.0728 (8)	0.0307 (5)	0.0416 (6)	−0.0135 (5)	−0.0179 (5)	−0.0005 (4)
Cl3	0.1158 (11)	0.0350 (6)	0.0486 (6)	−0.0027 (6)	−0.0209 (6)	−0.0222 (5)
Cl1	0.0801 (9)	0.0472 (7)	0.0746 (8)	−0.0293 (6)	−0.0338 (7)	0.0168 (6)
Cl2	0.1151 (12)	0.0745 (9)	0.0677 (8)	0.0119 (8)	−0.0340 (8)	−0.0440 (7)
O3	0.0622 (18)	0.0227 (13)	0.0366 (14)	−0.0029 (12)	−0.0151 (13)	−0.0119 (11)
C5	0.034 (2)	0.0258 (19)	0.0303 (18)	−0.0034 (15)	−0.0046 (16)	−0.0087 (15)
O5	0.073 (2)	0.0312 (15)	0.0346 (16)	−0.0132 (15)	−0.0142 (15)	−0.0038 (14)
N2	0.0419 (19)	0.0222 (16)	0.0315 (16)	−0.0044 (13)	−0.0051 (14)	−0.0090 (13)
N1	0.0420 (18)	0.0271 (17)	0.0326 (16)	−0.0042 (14)	−0.0059 (14)	−0.0104 (13)
C9	0.058 (3)	0.026 (2)	0.039 (2)	−0.0008 (18)	−0.014 (2)	−0.0011 (17)
O2	0.0396 (17)	0.0558 (19)	0.0453 (16)	0.0073 (15)	−0.0018 (14)	0.0063 (14)
C13	0.037 (2)	0.029 (2)	0.037 (2)	−0.0090 (17)	−0.0027 (18)	−0.0108 (17)
C16	0.041 (2)	0.0239 (19)	0.0350 (19)	−0.0038 (16)	−0.0062 (17)	−0.0094 (16)
C11	0.066 (3)	0.029 (2)	0.052 (2)	−0.010 (2)	−0.010 (2)	−0.018 (2)
C15	0.041 (2)	0.024 (2)	0.034 (2)	−0.0025 (16)	−0.0069 (17)	−0.0084 (16)
O4	0.113 (3)	0.0235 (15)	0.0449 (16)	0.0005 (16)	−0.0331 (17)	−0.0086 (13)
C14	0.032 (2)	0.030 (2)	0.039 (2)	−0.0029 (16)	−0.0048 (17)	−0.0088 (16)
C1	0.060 (3)	0.024 (2)	0.044 (2)	−0.0006 (19)	−0.018 (2)	−0.0048 (17)
C12	0.072 (3)	0.043 (3)	0.051 (3)	−0.018 (2)	−0.008 (2)	−0.026 (2)
C8	0.051 (3)	0.0200 (19)	0.047 (2)	−0.0035 (17)	−0.007 (2)	−0.0049 (17)
C10	0.051 (2)	0.030 (2)	0.035 (2)	−0.0034 (18)	−0.0145 (18)	−0.0065 (17)
C6	0.034 (2)	0.0242 (19)	0.0298 (18)	−0.0039 (15)	0.0009 (15)	−0.0097 (15)
C7	0.040 (2)	0.0229 (19)	0.042 (2)	−0.0055 (16)	−0.0010 (18)	−0.0088 (17)
C4	0.044 (2)	0.039 (2)	0.036 (2)	−0.0094 (18)	−0.0024 (18)	−0.0149 (18)
C2	0.063 (3)	0.043 (3)	0.040 (2)	−0.005 (2)	−0.020 (2)	−0.006 (2)
O1	0.0491 (19)	0.083 (2)	0.0386 (16)	0.0057 (16)	−0.0020 (15)	−0.0158 (16)
C3	0.057 (3)	0.052 (3)	0.038 (2)	−0.010 (2)	−0.014 (2)	−0.016 (2)

### Geometric parameters (Å, °)

**Table:**

Cu1—O3	1.939 (2)	C13—O1	1.213 (4)
Cu1—O5	1.971 (2)	C13—C14	1.536 (5)
Cu1—N1	1.994 (3)	C16—C15	1.531 (5)
Cu1—N2	2.027 (3)	C16—H16	0.9800
Cu1—O1	2.152 (3)	C11—C12	1.359 (6)
Cl4—C16	1.774 (3)	C11—C7	1.417 (5)
Cl3—C16	1.759 (4)	C11—H11	0.9300
Cl1—C14	1.767 (4)	C15—O4	1.223 (4)
Cl2—C14	1.753 (4)	C14—H14	0.9800
O3—C15	1.261 (4)	C1—C2	1.374 (5)
C5—N1	1.348 (4)	C1—H1	0.9300
C5—C4	1.394 (5)	C12—C4	1.432 (5)
C5—C6	1.444 (5)	C12—H12	0.9300
O5—H5A	0.85 (3)	C8—C7	1.410 (5)
O5—H5B	0.85 (3)	C8—H8	0.9300
N2—C10	1.331 (4)	C10—H10	0.9300
N2—C6	1.355 (4)	C6—C7	1.401 (5)
N1—C1	1.348 (4)	C4—C3	1.411 (5)
C9—C8	1.355 (5)	C2—C3	1.349 (5)
C9—C10	1.399 (5)	C2—H2	0.9300
C9—H9	0.9300	C3—H3	0.9300
O2—C13	1.240 (5)		
			
Cl1···Cl4^i^	3.306 (2)	Cg1···Cg2^iii^	3.734 (2)
Cl2···Cl3^ii^	3.278 (2)		
			
O3—Cu1—O5	91.06 (10)	O4—C15—O3	127.3 (3)
O3—Cu1—N1	171.76 (11)	O4—C15—C16	117.8 (3)
O5—Cu1—N1	95.86 (11)	O3—C15—C16	114.7 (3)
O3—Cu1—N2	90.28 (11)	C13—C14—Cl2	110.9 (3)
O5—Cu1—N2	149.53 (12)	C13—C14—Cl1	109.3 (2)
N1—Cu1—N2	81.49 (11)	Cl2—C14—Cl1	110.0 (2)
O3—Cu1—O1	95.15 (11)	C13—C14—H14	108.9
O5—Cu1—O1	101.24 (12)	Cl2—C14—H14	108.9
N1—Cu1—O1	87.88 (12)	Cl1—C14—H14	108.9
N2—Cu1—O1	108.94 (12)	N1—C1—C2	122.1 (4)
C15—O3—Cu1	127.3 (2)	N1—C1—H1	118.9
N1—C5—C4	124.2 (3)	C2—C1—H1	119.0
N1—C5—C6	115.7 (3)	C11—C12—C4	121.4 (4)
C4—C5—C6	120.1 (3)	C11—C12—H12	119.3
Cu1—O5—H5A	99 (2)	C4—C12—H12	119.3
Cu1—O5—H5B	137 (3)	C9—C8—C7	119.6 (3)
H5A—O5—H5B	111 (3)	C9—C8—H8	120.2
C10—N2—C6	117.7 (3)	C7—C8—H8	120.2
C10—N2—Cu1	129.8 (3)	N2—C10—C9	121.9 (4)
C6—N2—Cu1	112.5 (2)	N2—C10—H10	119.0
C1—N1—C5	117.1 (3)	C9—C10—H10	119.1
C1—N1—Cu1	128.6 (3)	N2—C6—C7	124.1 (3)
C5—N1—Cu1	114.1 (2)	N2—C6—C5	116.1 (3)
C8—C9—C10	120.4 (4)	C7—C6—C5	119.8 (3)
C8—C9—H9	119.8	C6—C7—C8	116.3 (3)
C10—C9—H9	119.8	C6—C7—C11	118.9 (3)
O1—C13—O2	125.7 (4)	C8—C7—C11	124.7 (3)
O1—C13—C14	117.0 (3)	C5—C4—C3	116.3 (3)
O2—C13—C14	117.3 (3)	C5—C4—C12	118.5 (3)
C15—C16—Cl3	113.0 (3)	C3—C4—C12	125.2 (3)
C15—C16—Cl4	112.4 (2)	C3—C2—C1	120.8 (3)
Cl3—C16—Cl4	109.30 (19)	C3—C2—H2	119.6
C15—C16—H16	107.3	C1—C2—H2	119.6
Cl3—C16—H16	107.3	C13—O1—Cu1	144.6 (3)
Cl4—C16—H16	107.3	C2—C3—C4	119.4 (3)
C12—C11—C7	121.2 (4)	C2—C3—H3	120.3
C12—C11—H11	119.4	C4—C3—H3	120.3
C7—C11—H11	119.4		

Symmetry codes: (i) −*x*+1, −*y*+2, −*z*; (ii) −*x*+1, −*y*+1, −*z*; (iii) −*x*, −*y*+1, −*z*+2.

### Hydrogen-bond geometry (Å, °)

**Table:**

*D*—H···*A*	*D*—H	H···*A*	*D*···*A*	*D*—H···*A*
C16—H16···O2^iv^	0.98	2.24	3.118 (5)	149
O5—H5B···O2^v^	0.85 (2)	1.81 (2)	2.654 (3)	174 (3)
O5—H5A···O4	0.85 (2)	1.86 (2)	2.673 (4)	159 (3)

Symmetry codes: (iv) *x*−1, *y*, *z*; (v) −*x*+1, −*y*+1, −*z*+1.
